# Predictors of shunt responsiveness and outcomes in idiopathic normal pressure hydrocephalus: a retrospective cohort study

**DOI:** 10.1007/s00701-026-06839-x

**Published:** 2026-03-23

**Authors:** Dror Shir, Noa Bregman, Jonathan Roth, Elissa Ash, Tamara Shiner

**Affiliations:** 1 Cognitive Neurology Unit, Neurological Institute, Tel Aviv Medical Center, Weizmann 6, Tel Aviv, 6423906 Israel; 2 Gray Faculty of Medical and Health Sciences, Tel Aviv University, Chaim Levanon St 55, Tel Aviv-Yafo, 6997801 Israel; 3 Department of Neurosurgery, Tel Aviv Medical Center, Weizmann 6, Tel Aviv, 6423906 Israel

**Keywords:** Normal Pressure Hydrocephalus (NPH), Shunt surgery, CSF tau, Clinical outcomes

## Abstract

**Background:**

Idiopathic Normal Pressure Hydrocephalus (iNPH) remains a challenging clinical diagnosis with variable treatment response.

**Objectives:**

This study aimed to identify clinical, imaging, and CSF biomarkers associated with favorable outcomes following shunt placement.

**Methods:**

All patients evaluated for iNPH at the Tel-Aviv Medical Center (TLVMC) between 2020 and 2022 were included. Participants underwent clinical, cognitive, and imaging assessments, high-volume lumbar puncture (LP). LP responders were referred for shunt placement, and outcomes assessed at one year.

**Results:**

183 patients were evaluated; 167 met criteria for suspected iNPH and underwent LP. Sixty-two (37%) patients showed improvement after CSF drainage and were referred for shunting. Of these, 38 (61%) underwent shunt placement. Gait disturbance was the most common presenting symptom (68%), and more frequent in LP responders (*p* = 0.007), whereas cognitive symptoms were more common among non-responders (29.5% vs. 10%). LP responders had lower CSF total tau (t-tau) (222.6 ± 99.1 vs. 256 ± 107.1, *p* = 0.045) and protein (41.1 ± 17.1 vs. 49.1 ± 25.7, *p* = 0.032) and were more likely to exhibit a disproportionately enlarged subarachnoid-space hydrocephalus (DESH) pattern (73% vs. 46%, *p* < 0.001). Among the 38 shunted patients, 21 (55%) had a favourable outcome at one year, which was associated with lower t-tau (*p* = 0.056) and more frequent DESH (*p* = 0.026).

**Conclusions:**

Over half of patients who underwent shunt placement experienced favorable clinical outcomes at one year. Lower t-tau and DESH pattern were associated with better outcomes.

## Introduction

Idiopathic Normal Pressure Hydrocephalus (iNPH), first described by Hakim and Adams in 1965 [16], is characterized by a triad of gait disturbance, cognitive impairment, and urinary incontinence. Its estimated prevalence in adults over 65 is ~ 0.5% [6, 41], though diagnosis is challenging due to symptom overlap with other age-related disorders.

iNPH is increasingly conceptualized as a multifactorial clinical-pathophysiological syndrome involving altered cerebrospinal fluid (CSF) dynamics, cerebrovascular factors, and coexisting neurodegenerative processes. Disruption of CSF circulation, characterized by ventricular enlargement, increased outflow resistance, and mildly elevated intracranial pressure, remains a central feature [27, 40]. Proposed mechanisms include impaired CSF reabsorption due to arachnoid villi dysfunction [10], venous pressure alterations [3, 5], mechanical compression from enlarged ventricles [5] and interactions with cerebrovascular disease [27]. Emerging data also suggest that glymphatic dysfunction and reduced arterial pulsatility may impair periventricular fluid exchange and protein clearance, potentially contributing to overlapping neurodegenerative pathology [38, 46].

Despite uncertainty regarding the precise pathophysiology, the standard treatment is CSF shunting [27]. Reported response rates following shunting vary widely (50–90%) in the first year after surgery [11, 23, 28, 36], with gait being the symptom most likely to improve [10, 33]. This variability likely reflects heterogeneity in patient selection [37].Clinical diagnosis of iNPH relies on the presence of the classic symptom triad (i.e., impairments in gait, cognition and urinary control), together with imaging evidence of ventriculomegaly not fully explained by atrophy [27]. Probable iNPH is typically defined by progressive symptoms, gait impairment plus at least one additional triad feature, and supportive neuroimaging findings [37]. Gait disturbance is usually the earliest and most prominent manifestation [1, 4, 28].

Characteristic imaging features include ventriculomegaly disproportionate to cortical atrophy and the disproportionately enlarged subarachnoid-space hydrocephalus (DESH) [22]. Although measures such as Evans index, callosal angle, and temporal horn enlargement are associated with shunt responsiveness, their specificity and negative predictive value remain limited [9, 13, 21, 44].

While gait often improves after shunting, long-term benefit varies and tends to decline over time. Untreated patients, who do not undergo shunting, tend to experience progressive decline and increased care dependency within five years [39]. The CSF tap test remains the most commonly used tool to predict surgical responsiveness, yet its sensitivity and specificity are highly variable (26–87%) [32, 45], and no single biomarker reliably identifies patients most likely to benefit [24].

In light of the above, we aimed to identify parameters predictive of shunt responsiveness. Our objectives were to describe the diagnostic processes, characterize features associated with favourable shunt outcomes, and assess outcomes in patients evaluated for suspected iNPH at TLVMC.

## Materials and methods

### Study design and population

This retrospective study included patients evaluated for suspected iNPH at the Neurology outpatient hospitalization unit of TLVMC between January 2020 and December 2022. The study period was selected to allow sufficient longitudinal follow-up of patients who underwent shunt placement. The study protocol was approved by the Tel-Aviv Medical Center IRB committee (IRB TLV-0427–24) in accordance with the Declaration of Helsinki and Israeli Ministry of Health regulations. The requirement for informed consent was waived due to the retrospective nature of the study.

### Data collection

Clinical data was extracted from electronic medical records and included demographic information, past medical history, and results of neurologic and cognitive assessments. Cognitive screening tools, including the Mini-Mental State Examination (MMSE) [25] and/or Montreal Cognitive Assessment (MoCA) [34] were recorded when available. Medical records were reviewed to identify individuals who met the diagnostic criteria for probable, possible, or unlikely iNPH, based on established international guidelines [37]. Other neurologic symptoms and signs were recorded, including Lewy body disease core features other than parkinsonism (visual hallucinations, cognitive fluctuations, or a history of REM sleep behavior disorder) [43].

### CSF tap test and clinical assessment

All patients underwent a high-volume LP with removal of at least 30 mL of CSF. Standardized evaluations of gait and cognition were conducted prior to lumbar puncture. The (MoCA) [34], administered by a neurologist was used to evaluate cognition, with a score below 26/30 indicating cognitive impairment. Gait was assessed using the Timed Up and Go (TUG) test, in which patients were timed as they rose from a chair, walked 6 m, turned, and returned to a seated position. Gait speed and step count were recorded manually by a trained neurologist. The TUG test was performed three times, and the average time and step count were calculated. Gait assessments were conducted at three time points: prior to LP, two hours post-LP, and 24 h post-LP. Percentage improvement in gait speed was calculated by subtracting the baseline gait speed from the post-LP gait speed, dividing the result by the baseline gait speed, and multiplying by 100. Clinical response to the LP was defined using predefined criteria [14]. Patients were classified as LP responders if they demonstrated either: (1) ≥ 10% improvement in gait speed on the TUG test two hours following CSF removal, or (2) clear physician-documented improvement in gait, balance, or functional mobility based on standardized neurological examination and patient/caregiver report. This cutoff was chosen based on prior literature and clinical practice standards indicating that ≥ 10% gait speed improvement reflects meaningful clinical change in iNPH. CSF-derived data, including protein and total tau (t-tau) levels, were recorded. The normal reference range for CSF protein is 15–45 mg/dL. T-tau levels were interpreted according to established thresholds: < 290 pg/mL (negative), 290–452 pg/mL (borderline), and > 452 pg/mL (positive).

### Imaging assessment

For participants with available brain MRI scans, a blinded imaging review was conducted by an experienced neurologist (DS). This review was performed independently, separate from the clinical medical record review, to minimize bias. All patients had formal clinical imaging reports issued by board-certified neuroradiologists as part of routine care, and these reports were reviewed in parallel. The purpose of the reassessment was to obtain uniform quantitative measurements (Evans Index and callosal angle) and to systematically assess for DESH features using predefined criteria to ensure methodological consistency across cases. Evans Index was measured on axial T1-weighted images, defined as the ratio of the maximum width of the frontal horns of the lateral ventricles to the maximum internal diameter of the skull at the same level, as previously described [13]. The callosal angle was assessed on coronal T1-weighted images perpendicular to the anterior–posterior commissure line, at the level of the posterior commissure, using standard measurement techniques [21]. White matter hyperintensities were rated using the Fazekas scale on axial T2-weighted FLAIR images, with scores assigned as none/mild, moderate, or severe. The presence of DESH was assessed dichotomously (yes/no), based on previously established imaging criteria, including tight high convexity sulci, enlarged Sylvian fissures, and ventriculomegaly [31].

### Diagnostic classification according to american-european guidelines

Each patient in the cohort was classified as having probable, possible, or unlikely iNPH based on the American-European consensus criteria [37]. The classification was made retrospectively using available clinical and imaging data. Patients were categorized as probable iNPH if they exhibited gait or balance disturbance, along with at least one additional symptom from the clinical triad (cognitive impairment and/or urinary incontinence or urgency). Additionally, brain imaging was required to show ventriculomegaly, defined as an Evans Index (EI) > 0.3, along with at least one of the following supportive imaging features: (a) narrow callosal angle, (b) enlargement of the temporal horns, or (c) periventricular signal changes not attributable to ischemic changes or demyelination. Patients were categorized as possible iNPH if they exhibited either (a) urinary incontinence and/or cognitive impairment in the absence of gait disturbance, or (b) gait disturbance alone, in both cases accompanied by ventriculomegaly (EI > 0.3) on brain imaging, but without the additional supportive imaging findings required for the probable category. Patients were classified as unlikely iNPH if they did not meet any of the above clinical criteria, or if symptoms were better explained by another diagnosis, or if there was no evidence of ventriculomegaly on imaging.

### Shunt referral and follow-up

Patients who demonstrated a clinically meaningful improvement following CSF drainage were classified as LP responders and referred for shunt surgery. Although LP responsiveness was the primary criterion for shunt referral, four patients were shunted despite not meeting response criteria, based on neurosurgical judgment. These patients were included in the LP non-responder group for analysis to maintain consistency with our primary classification method. Shunt outcome at one year was defined as positive if there was sustained improvement in one or more domains (gait, cognition, or activities of daily living) without clinical decline in other domains, as documented in neurological assessment and patient/caregiver reports. This approach aligns with established criteria used in clinical trials and guideline-supported practice [17].

### Statistical analysis

Descriptive statistics were used to summarize cohort characteristics. Continuous variables were compared using independent-samples t-tests. Categorical variables were compared using Chi-square tests or Fisher’s exact tests. A binary logistic regression analysis was conducted to evaluate predictors of favorable shunt response at one year, including age at symptom onset, gender, and CSF total tau concentration as covariates. A *p*-value < 0.05 was considered statistically significant. Analyses were performed using SPSS (Version 28.0. Armonk, NY).

## Results

### Cohort overview

**Fig. 1:**
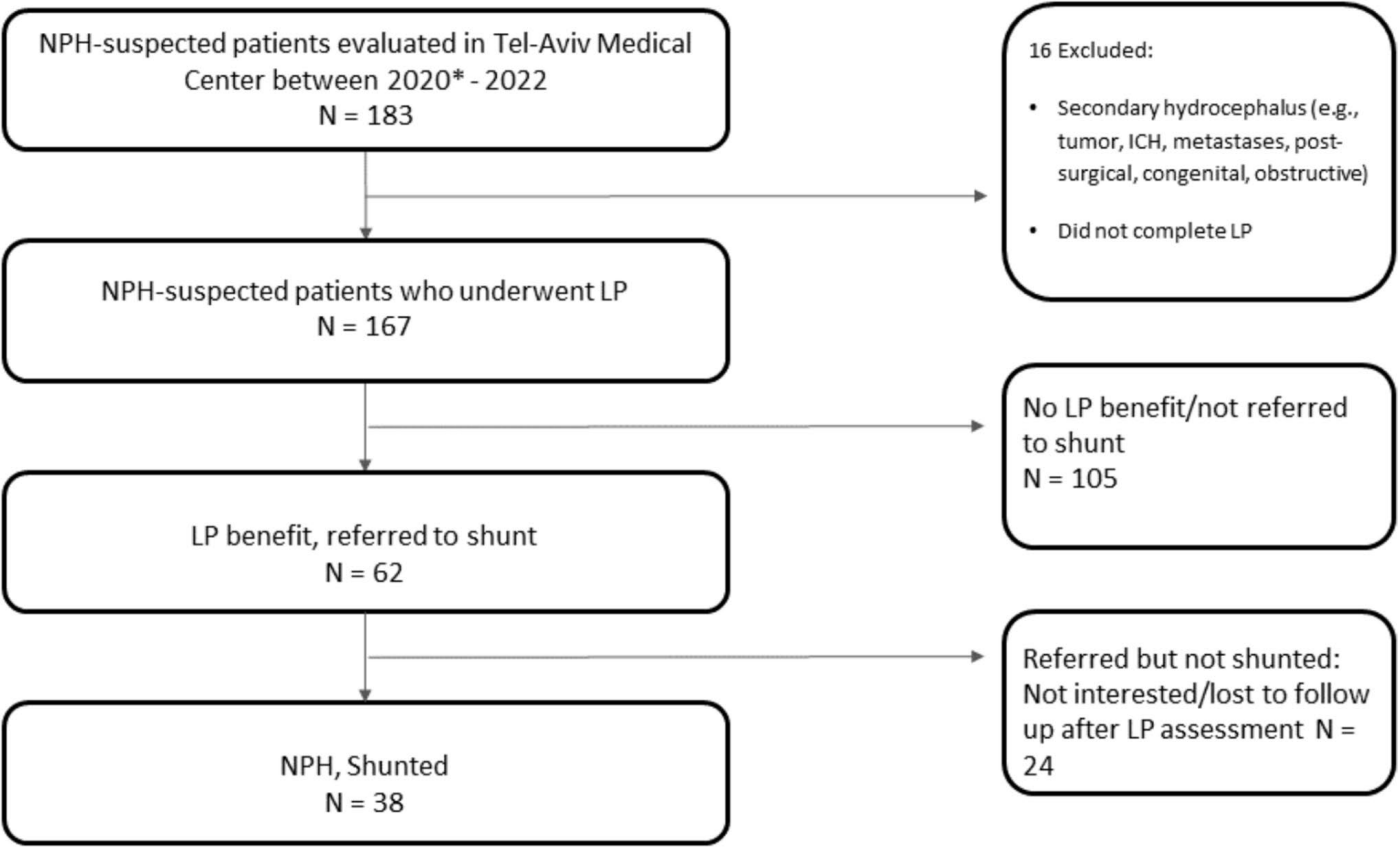
Study cohort flow diagram. Flowchart illustrating the inclusion process of patients evaluated for suspected iNPH at Tel-Aviv Medical Center between 2020 and 2022. Abbreviations: NPH: normal pressure hydrocephalus; ICH: intracranial hemmorhage; LP: lumbar puncture

A total of 183 patients were evaluated for suspected iNPH at Tel-Aviv Medical Center between 2020 and 2022. After excluding 16 patients due to secondary causes of hydrocephalus or incomplete LP assessment, 167 patients underwent LP and were included in the final analysis (Fig. 1). Of the 167 patients who underwent LP, 62 patients (37%) demonstrated a clinical benefit from CSF drainage and were referred for shunt surgery. Of these, 38 patients (61% of the responders) proceeded to shunt placement, while the remaining 24 (39% of the responders) declined surgery, primarily due to patient choice.

### Comparison between LP-responders and LP non-responders

Table 1 summarizes demographic characteristics, clinical variables, imaging findings, and fluid biomarker data for the study cohort. More male patients were assessed for iNPH (63%) compared to female patients. Mean age at symptom onset was 73.1 ± 6.6. Time from symptom onset to LP assessment had a mean of 33.3 ± 6.6 months (2.8 years). Mean MOCA score of study participants was 17.9 ± 5.8.

**Table 1:** Patient characteristics according to LP response

	Full cohort	LP responsive (*n* = 62)	LP non-responsive (*n* = 105)	*P* value
Female (%)	62 (37%)	22 (35.5%)	40 (38%)	0.868
Hypertension	111 (66.5%)	32 (56%)	76 (72%)	**0.022**
Hyperlipidemia	90 (54%)	30 (48%)	60 (57%)	0.251
Ischemic heart disease	42 (25%)	10 (16%)	32 (30%)	**0.041**
Diabetes	76 (45.5%)	29 (47%)	47 (45%)	0.872
Age at symptom onset	73.1 ± 6.6	71.6 ± 5.6	74.1 ± 7.0	**0.018**
Time from symptom onset to assessment (months)	33.3 ± 25.3	34.9 ± 25.8	32.3 ± 25.1	0.536
MOCA (/30)	17.9 ± 5.8	18 ± 4.9	17.9 ± 6.2	0.901
Mean walk test time, 6 m, before LP (seconds)	27.7 ± 13.7	28.9 ± 13.2	26.9 ± 13.9	0.397
Mean walk test time, 6 m, 2 h after LP (seconds)	25.3 ± 11.4	23.7 ± 8.7	26.3 ± 12.6	0.17
Percent speed improvement	5.9 ± 16.8	14.6 ± 12.8	0.85 ± 16.9	** < 0.001**
Mean number of steps (6 m), before LP	15.6 ± 5.2	17.4 ± 5.6	14.4 ± 4.6	** < 0.001**
Mean number of steps (6 m), 2 h after LP	14.9 ± 4.6	15.3 ± 4.8	14.6 ± 4.5	**0.0206**
Mean numbers of steps per turn, before LP	4.5 ± 2.2	5.2 ± 2.4	4.1 ± 2.0	**0.002**
Mean numbers of steps per turn, 2 h after LP	3.7 ± 1.6	3.8 ± 1.6	3.7 ± 1.6	**0.0409**
CSF protein levels	46.0 ± 23.1	41.1 ± 17.1	49.1 ± 25.7	**0.032**
CSF tau levels	243.3 ± 104	222.6 ± 99.1	256 ± 107.1	**0.045**
Evans Index	0.4 ± 0.4	0.3 ± 0.04	0.5 ± 0.6	0.092
Callosal angle	99.13 ± 20.9	96.4 ± 18.1	101.5 ± 22.94	0.129
DESH pattern	53 (58%)	30 (73%)	23 (46%)	** < 0.001**
Fazekas score*				
None/Mild	50 (55%)	25 (61%)	25 (50%)	0.102
Moderate	23 (25%)	6 (15%)	17 (34%)	
Severe	18 (20%)	10 (24%)	8 (16%)	

T-Test for continuous variables, Chi-square for categorical comparisons

For imaging measures, *n* = 91. Cut-off values: Evans index > 0.3, callosal angle < 90°

For CSF protein, *n* = 163. For CSF TTAU, *n* = 115

*CSF total tau reference levels: negative < 290 pg/ml; borderline 290–452 pg/ml; positive > 452 pg/ml

**For Fazekas scores: Mild: 0, 0–1, 1; Moderate: 1–2, 2; Severe 2–3, 3

**Fig. 2:**
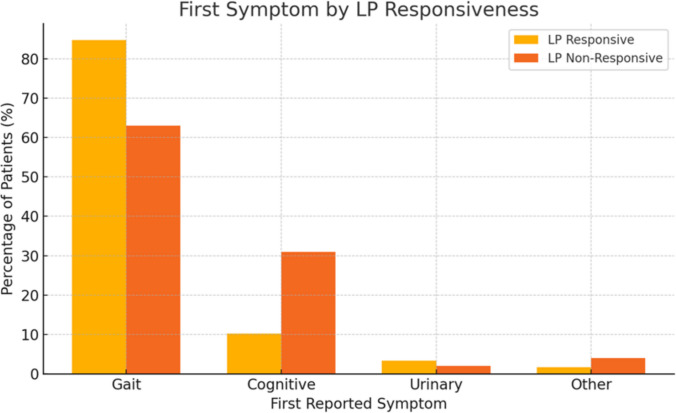
Distribution of first reported symptom by LP responsiveness. Bar graph showing the percentage of patients reporting gait, cognitive, urinary, or other symptoms as their first presenting symptom, stratified by lumbar puncture (LP) responsiveness. Other symptoms include: behavioral change, headaches, depression

Among the 167 patients evaluated, 62 (37%) were classified as LP responders. The most common presenting symptom in the cohort was gait disturbance, reported in 113 of 167 patients (68%). LP responders were significantly more likely to present with gait disturbance as the initial symptom compared to LP non-responders (*p* = 0.007, Fig. 2). In contrast, cognitive symptoms were reported as the first symptom in 31 of 105 non-responders (29.5%) versus only 6 of 62 LP responders (10%). LP responders had a lower prevalence of hypertension (56% vs. 72%, *p* = 0.022) and ischemic heart disease (16% vs. 30%, *p* = 0.041) compared to non-responders. In contrast, there were no significant group differences in sex distribution or in the prevalence of other vascular risk factors or comorbidities, including diabetes, hyperlipidemia, obstructive sleep apnea and chronic kidney disease.

LP responders were younger at symptom onset (71.6 ± 5.6 vs. 74.1 ± 7.0 years, *p* = 0.018), but time from symptom onset to assessment and baseline MOCA scores were similar between groups. LP responders exhibited significantly greater baseline gait impairment, taking more steps to walk 6 m (17.4 ± 5.6 vs. 14.4 ± 4.6, *p* < 0.001) and requiring more steps to complete a turn (5.2 ± 2.4 vs. 4.1 ± 2.0, *p* = 0.002). As expected, LP responders demonstrated a greater functional improvement in gait speed post-LP, reflecting enhanced responsiveness to CSF drainage (14.6 ± 12.8% vs. 0.85 ± 16.9%, *p* < 0.001). Two hours after LP, both measures improved more markedly in LP-responders, reflecting a functional improvement in gait pattern, with reductions in the number of steps over 6 m (15.3 ± 4.8 vs. 14.6 ± 4.5, *p* = 0.0206) and number of steps per turn (3.8 ± 1.6 vs. 3.7 ± 1.6, *p* = 0.0409).

**Fig. 3:**
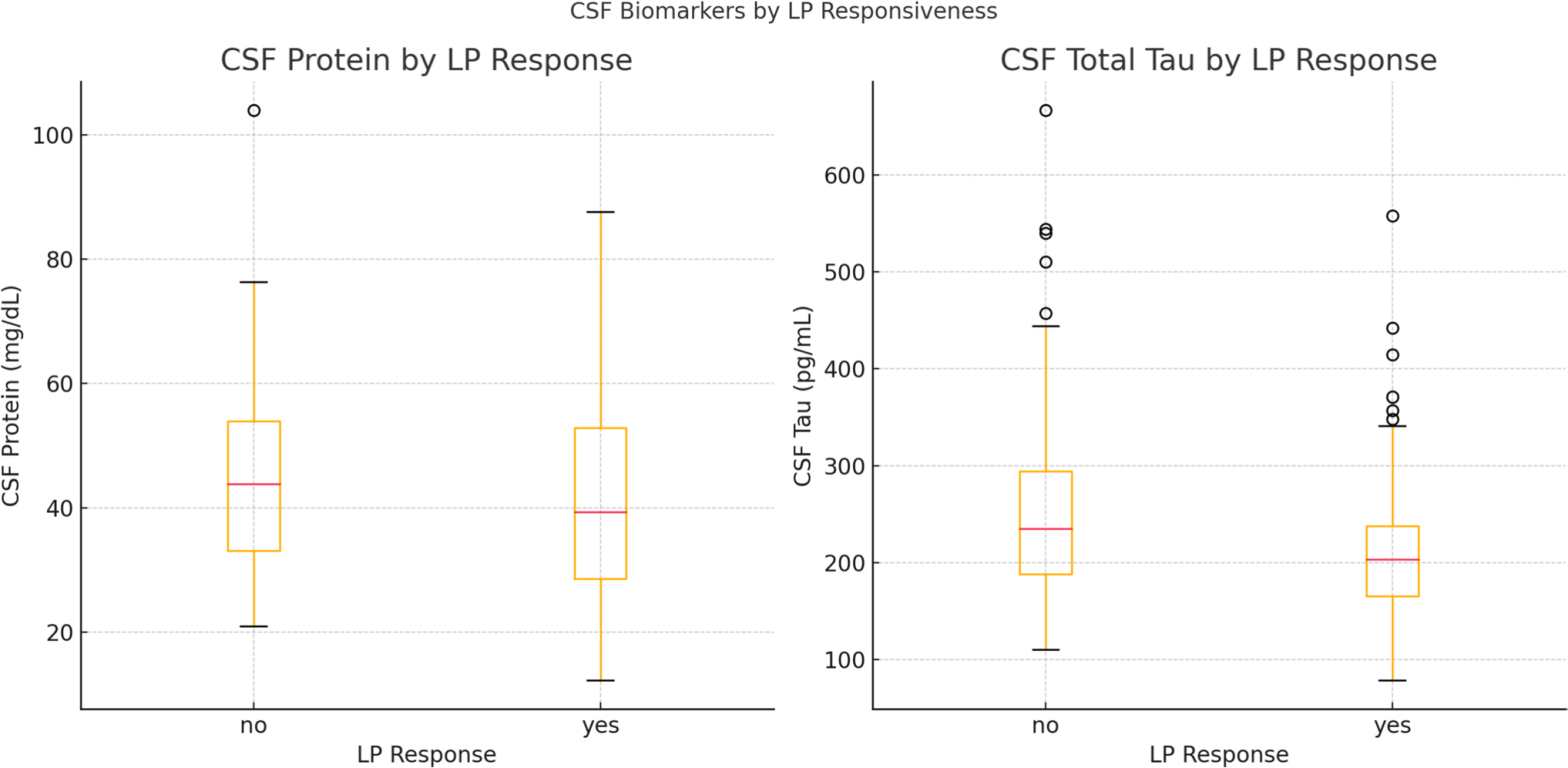
Boxplots illustrating cerebrospinal fluid (CSF) protein concentration (left) and total tau levels (right) in patients stratified by clinical response to high-volume lumbar puncture (LP). Patients classified as LP responders (*n* = 62) demonstrated lower CSF protein (*p* = 0.032) and total tau levels (*p* = 0.045) compared to non-responders (*n* = 105). Boxes represent the interquartile range (IQR), horizontal lines indicate medians, and whiskers extend to 1.5 × IQR

CSF protein and tau levels were lower in LP responders compared to non-responders (protein: 41.1 ± 17.1 vs. 49.1 ± 25.7 mg/dL, *p* = 0.032; tau: 222.6 ± 99.1 vs. 256 ± 107.1 pg/mL, *p* = 0.045, Fig. 3). The DESH pattern was more commonly observed in responders (73% vs. 46%, *p* < 0.001). While there were no statistically significant differences in Evans index or callosal angle, a trend was noted toward a smaller Evans index and narrower callosal angle in the LP-responsive group. Fazekas score distributions did not differ significantly between groups (*p* = 0.102).

There was no significant difference between groups in the presence of clinical features suggestive of Lewy body disease (LBD). Nineteen patients exhibited at least one core LBD feature other than parkinsonism, such as visual hallucinations, cognitive fluctuations, or a history of REM sleep behavior disorder, of whom 14 were classified as LP non-responders. However, this difference in frequency did not reach statistical significance.

A significant association was observed between diagnostic certainty and referral for shunt placement. Among patients classified as having probable iNPH, the majority (46/76, 60.5%) were considered LP responsive and referred for shunt, compared to only 16/86 (18.6%) of those considered possible iNPH, and none of the patients categorized as unlikely iNPH (0/5, *p* < 0.001).

### Patients who underwent shunt placement

**Fig. 4:**
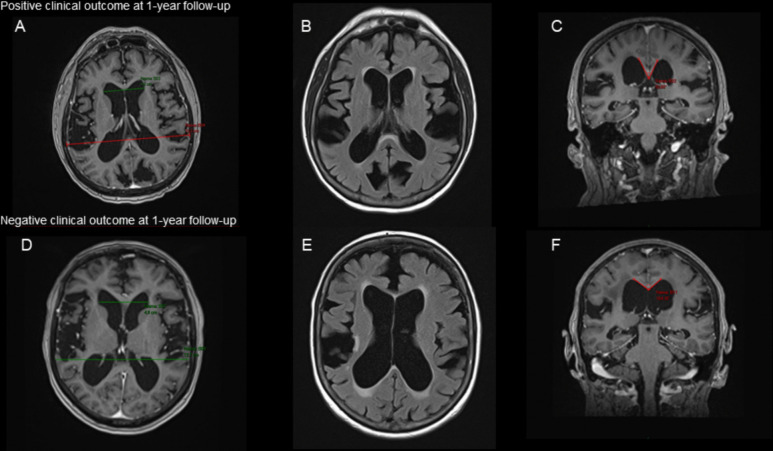
Brain MRI of two patients who underwent shunt placement, performed during pre-shunt clinical assessment. **A–C** Images from a patient with a positive clinical outcome at 1-year follow-up. **A** Axial T1-weighted image shows an Evans Index of 0.3409. **B** Axial FLAIR image shows mild white matter hyperintensities (Fazekas score 0). **C** Coronal T1-weighted image at the posterior commissure demonstrates a callosal angle of 52.65° and DESH pattern. **D–F** Images from a patient with a negative outcome at 1-year. **D** Axial T1-weighted image shows an Evans Index of 0.3701. **E** Axial FLAIR image shows more pronounced asymmetric white matter disease hyperintensities. **F** Coronal T1-weighted image at the posterior commissure demonstrates a callosal angle of 107.49°, DESH pattern was absent

Table 2 summarizes demographics, clinical variables, imaging and fluid biomarkers of the subgroup of patients who underwent shunt placement. Out of 167 patients evaluated for suspected iNPH, 38 underwent shunt placement. Among them, 21 (55%) experienced a favorable clinical outcome at one year. Baseline cognitive scores (MoCA) were similar between patients with a positive outcome and those with a negative outcome (18.3 ± 5.5 vs. 18.0 ± 4.8, *p* = 0.438). Those with positive outcomes were characterized by a greater immediate post-LP improvement in gait speed (percent speed improvement two hours post-LP, 21.8 ± 15.4%) compared to those with a negative outcome at one year (12.4 ± 12.04%, *p* = 0.027) and a higher prevalence of DESH on MRI (88% compared to 47% in those with a negative outcome, *p* = 0.026). Representative MRI findings are shown in Fig. 4, comparing imaging biomarkers between a patient with a positive outcome one-year post-shunting (top row) and a patient with a negative outcome (bottom row). Lower CSF t-tau levels also trended toward significance in predicting positive shunt response (lower levels in patients with a positive outcome at one year follow up: 191.5 ± 90.5 vs. 279.2 ± 127.7 pg/mL, *p* = 0.056). Other variables such as age, baseline cognitive scores, and standard MRI metrics (Evans Index, callosal angle) did not show a clear association with one-year outcomes.

**Table 2:** Patient Characteristics according to shunt outcome at one year

	All patients who underwent shunt (*n* = 38)	Positive shunt result, 1 year post procedure (*n* = 21)	Negative shunt result, 1 year post procedure (*n* = 17)	*P* value
Female (%)	14 (37%)	9 (43%)	5 (29%)	0.506
Hypertension	24 (63%)	14 (66%)	10 (59%)	0.74
Hyperlipidemia	17 (45%)	12 (57%)	5 (29%)	0.111
Ischemic heart disease	8 (21%)	5 (24%)	3 (18%)	0.709
Diabetes	19 (50%)	9 (53%)	10 (59%)	0.515
Age at symptom onset	71 ± 5.6	70.6 ± 3.7	71.0 ± 7.3	0.41
Time from symptom onset to assessment (months)	32.2 ± 24.6	34.9 ± 28.0	27.7 ± 19.6	0.196
MOCA (/30)	18 ± 5.1	18.3 ± 5.5	18.0 ± 4.8	0.438
Average walk test time, 6 m, before LP (seconds)	29.3 ± 11.6	31.6 ± 14.6	27.3 ± 7.3	0.142
Average walk test time, 6 m, 2 h after LP (seconds)	23.1 ± 5.6	22.9 ± 5.7	23.5 ± 5.8	0.39
Percent speed improvement	17.4 ± 14.3	21.8 ± 15.4	12.4 ± 12.04	**0.027**
Mean number of steps (6 m), before LP	17.45 ± 5.7	16.6 ± 3.8	18.4 ± 7.4	0.375
Mean number of steps (6 m), 2 h after LP	15.6 ± 5.3	14.6 ± 3.4	16.6 ± 6.7	0.310
Mean numbers of steps per turn, before LP	5.1 ± 1.9	5.4 ± 2.2	4.7 ± 1.5	0.379
Mean numbers of steps per turn, 2 h after LP	3.7 ± 1.2	3.6 ± 1.3	3.8 ± 1.1	0.800
CSF protein levels	38.9 ± 19.3	38.2 ± 18.8	40.4 ± 21.1	0.374
CSF tau levels	230.1 ± 114.9	191.5 ± 90.5	279.2 ± 127.7	0.056
Evans Index	0.3 ± 0.04	0.3 ± 0.04	0.4 ± 0.03	0.656
Callosal angle	95.6 ± 17.0	91.9 ± 18.4	101.7 ± 13.1	0.067
DESH pattern	23 (60%)	15 (88%)	8 (47%)	**0.026**
Fazekas score*				
None/Mild	19 (65%)	13 (72%)	6 (54%)	0.636
Moderate	5 (17%)	3 (17%)	2 (18%)	
Severe	5 (17%)	2 (11%)	3 (27%)	

Gait parameters refer to changes observed following the initial high-volume LP, prior to shunt surgery. Outcome status reflects clinical assessment at one-year follow-up post-shunt placement

T-Test for continuous variables, Fisher's exact for categorical comparisons

For imaging measures, *n* = 29. For CSF protein, *n* = 36 For CSF TTAU, *n* = 24. Cut-off values: Evans index > 0.3, callosal angle < 90°

*CSF total tau reference levels: negative < 290 pg/ml; borderline 290–452 pg/ml; positive > 452 pg/ml

**For Fazekas scores: Mild: 0, 0–1, 1; Moderate: 1–2, 2; Severe 2–3, 3

***1-year follow-up: Two patients had only 4- and 6-month follow-up post-shunt but were included based on a positive outcome at their last available assessment

### Additional biomarker observations

FDG-PET scans were performed on 19 patients, of whom 14 exhibited cortical hypometabolic patterns suggestive of an underlying neurodegenerative process. Among these, only three patients (21%) were classified as LP responders and referred for shunt placement. All three underwent the procedure, with two experiencing negative outcomes at one-year follow-up and one demonstrating clinical improvement. In contrast, among the five patients with normal FDG-PET findings, two were referred for shunting based on LP responsiveness; one had a positive post-shunt outcome, while the other did not improve. FDOPA-PET imaging was performed in 15 patients to assess presynaptic dopaminergic function. Eleven patients had normal FDOPA-PET scans, and five of these were referred for shunting based on a positive LP response. Four proceeded to shunt placement, all of whom experienced favorable outcomes at one-year follow-up. Of the four patients with abnormal FDOPA-PET findings, three were non-responsive to LP and were not considered for shunting. The remaining patient, despite demonstrating LP responsiveness, elected not to undergo the procedure.

## Discussion

Correctly identifying patients with suspected iNPH who stand to benefit from shunting is a key challenge for the medical teams to whom these patients present. In this single-center retrospective study of patients evaluated for suspected iNPH, we found that over half of patients who underwent shunting experienced a favourable outcome at one year. This rate is slightly lower than previously reported improvement rates, which range from 59 to 75% following shunt insertion [15, 18]. Critically, those who experienced a favourable outcome at one-year, tended to have lower CSF tau levels. They also showed a greater post-LP gait improvement compared to those with negative outcomes.

CSF total tau levels were lower in the LP-responsive patients compared to non-responders, with a similar trend observed among those who underwent shunt placement and had a favorable clinical outcome at one-year. While prior studies have shown that CSF t-tau levels are lower in iNPH compared to neurodegenerative disorders such as AD, only a limited number have explored its predictive value for shunt responsiveness. Craven et al. [8] reported that lower t-tau levels were associated with improved postoperative mobility in 79 patients diagnosed with iNPH, and a meta-analysis by Thavarajasingam et al. [42] found that lumbar CSF levels of both phosphorylated tau and total tau were significantly higher in shunt non-responsive iNPH compared to responsive cases. Our findings further support the role of t-tau as a potential prognostic biomarker with lower baseline t-tau levels associated with favorable clinical outcomes even one year following shunt surgery. Lower tau levels may reflect a subgroup with less advanced neurodegenerative burden, whose symptoms are more directly attributable to reversible CSF flow disturbances. LP responsiveness was also associated with younger age at symptom onset and fewer vascular comorbidities, suggesting that a less advanced or less comorbid disease state may enhance responsiveness to CSF drainage. This contrasts with prior reports identifying vascular disease as a risk factor for iNPH [7], likely reflecting differences in cohort selection and referral patterns. Vascular comorbidities may also attenuate measurable gait improvement, and our modest sample size limits firm conclusions regarding their independent contribution. We therefore interpret these findings cautiously and consider vascular disease a potential confounder rather than a strict exclusionary factor. LP responders also had more pronounced gait inefficiency at baseline, and were more likely to exhibit DESH on imaging, supporting the value of integrating clinical, imaging and CSF biomarker data to refine patient selection.

A greater proportion of men were evaluated for iNPH in our cohort. Although some studies have not identified sex differences in prevalence [29],larger brain volume, more common in men, has been proposed as a potential risk factor for iNPH [20]. Alternatively, the observed male predominance may reflect a referral bias. Sex distribution was similar among LP-responders and shunted patients, suggesting that sex may influence referral rates but not the likelihood of being selected for surgical treatment. Under-recognition of urinary symptoms in women, particularly in older populations, may also contribute to this imbalance [35].

The differential diagnosis for iNPH is broad, including cerebrovascular disease, AD, Parkinson's disease and other neurodegenerative disorders. Imaging biomarkers may aid diagnosis. Although only a small subset of our patients underwent FDG-PET, a neurodegenerative pattern appeared to be associated with lower LP responsiveness, consistent with prior studies showing that comorbid neurodegeneration (e.g., Alzheimer's disease) reduces shunt benefit [2, 19, 30, 40]. FDG-PET could thus contribute to preoperative risk stratification, though our findings are hypothesis-generating given the small sample size. Similarly, normal FDOPA-PET scans may predict better outcomes: among 11 patients with normal FDOPA-PET, all four who underwent shunting had a favorable outcome at one year, while most patients with abnormal FDOPA-PET were LP non-responders.

Even after the diagnosis of iNPH was made, 39% of LP-responsive patients ultimately declined shunt surgery. Several factors may have contributed to this, including concerns about surgical risks and complications, uncertainty regarding the durability or magnitude of benefit and personal preference to avoid invasive procedures [17, 26]. Even among patients who undergo shunt placement, clinical outcomes remain variable. Recent placebo-controlled evidence demonstrated significant improvement in gait velocity and balance measures at three months following shunting in appropriately selected patients [26], thereby strengthening the evidence base for surgical intervention. However, that study evaluated short-term outcomes only. In our cohort, although 55% of shunted patients experienced a favorable clinical outcome one year, 45% did not, highlighting the limitations of current diagnostic approaches in predicting sustained benefit. Our findings align with prior studies, which have demonstrated that while short-term improvements following shunting are common [17], clinical benefits often diminish over longer follow-up periods. For example, Espay et al. [12], that only 32% of patients maintained persistent post-shunt benefits at 36 months, with over 25% receiving revised diagnoses of underlying neurodegenerative diseases such as AD, LBD, or progressive supranuclear palsy. These observations support the hypothesis that in some cases, ventriculomegaly attributed to iNPH may represent early manifestations of neurodegenerative disorders rather than true idiopathic hydrocephalus. Our findings support this, as higher CSF t-tau levels may well be representing early neurodegeneration and may explain why those patients with unfavourable one-year outcomes had higher levels. Our results suggest that specific preoperative features, including greater gait improvement after CSF drainage and lower CSF tau levels, may predict better outcomes, whereas standard clinical variables such as age, sex, vascular comorbidities, and baseline cognitive performance may not differentiate responders from non-responders. Imaging markers also played a role: the presence of a DESH pattern on MRI was significantly associated with a favorable outcome, while Evans Index, callosal angle, and white matter burden did not.

Several limitations of our study should be acknowledged. First, the retrospective design introduces inherent risks of selection bias, information bias, and incomplete data capture. Shunt placement was offered predominantly to patients who exhibited a clinical response following high-volume lumbar puncture. Therefore, comparison of shunt outcomes between LP responders and non-responders was not possible, potentially limiting conclusions regarding the predictive value of the tap test for surgical outcomes. Reliance on LP responsiveness as a strict prerequisite for surgical referral therefore carries a potential risk of undertreatment. Second, the sample size, particularly the subgroup of patients who underwent shunt placement and had biomarker analyses (e.g., CSF tau levels, PET imaging), was relatively small, limiting the power to detect more subtle associations. Although we followed a standardized diagnostic protocol, assessments such as gait improvement and clinical impressions, determination of LP responsiveness was partially subjective and may vary between evaluators. Another limitation is that longitudinal postoperative outcomes were based on routine clinical assessments and physician judgment rather than a standardized quantitative scale, which may introduce subjective variability. Finally, iNPH is a heterogeneous clinical syndrome that frequently coexists with neurodegenerative disease [2, 40]. While the retrospective nature and modest sample size constrained us to simpler statistical methods, we acknowledge that more advanced approaches, such as multinomial logistic regression or mixed-effects models, would better account for overlapping pathologies and enhance the robustness of predictive modelling. We recommend these methods for future work in larger, prospective cohorts.

## Conclusion

In this real-world cohort, our findings highlight the complex and multifactorial nature of diagnosing and prognosticating outcomes in iNPH and the challenges inherent in predicting sustained benefit from shunt surgery. In our sample, over half of shunted patients experienced favorable outcomes at one year. Lower CSF total tau levels and the presence of DESH were associated with improved outcomes. Further research is needed to refine risk stratification, including the integration of additional biomarkers of neurodegeneration. Ultimately, our results support a multimodal approach that combines clinical response to CSF drainage with imaging and fluid biomarkers to improve diagnostic precision and optimize decision-making in patients with suspected iNPH.

## Data Availability

The data that support the findings of this study are available from the corresponding author, TS, upon reasonable request.
